# THE EFFECTS OF TYPE 2 DIABETES MELLITUS ON POSTURAL ADAPTATION DURING A VISUAL SEARCH TASK IN OLDER ADULTS

**DOI:** 10.1093/geroni/igac059.2874

**Published:** 2022-12-20

**Authors:** Alexander Wolfe, Tongjian You, Suzanne Leveille, Brad Manor, Azizah Jor’dan

**Affiliations:** University of Massachusetts Boston, Dorchester, Massachusetts, United States; University of Massachusetts Boston, Boston, Massachusetts, United States; University of Massachusetts Boston, Boston, Massachusetts, United States; Hebrew SeniorLife/Harvard Medical School, Roslindale, Massachusetts, United States; University of Massachusetts, Boston, Massachusetts, United States

## Abstract

Type II diabetes mellitus (T2DM) is associated with a reduction in sensory integration capacity that often results in cognition and postural control deficits. The effects of T2DM on the ability to adapt one’s postural sway while standing and performing a visual search task (VST) are unknown. Twenty-three healthy older adults (HOA) (70–90 years) and 20 older adults with T2DM (67–93 years) performed multiple trials of quiet standing with and without a VST (i.e., counting 1 target letter in a grid of random letters). Postural sway acceleration measures were jerk, velocity, range, and pathlength in the anterior-posterior (AP) and medial-lateral (ML) direction, as well as elliptical area. Postural adaptation to the VST was defined as the absolute change between conditions. VST accuracy (%) was defined as the percentage of participant-reported target letters compared to total target letters. In general, for both groups, performing the VST resulted in an average reduction in sway jerk, velocity, pathlength, and range when performing the VST, compared to control (T2DM & HOA, p < 0.04). VST accuracy and the magnitude of postural adaptation to the VST were similar between groups (p>0.15). Within the T2DM group, those who performed worse on the VST exhibited less adaptation (i.e., smaller decrease) in ML velocity (r=0.47, p=0.04). No other differences or associations were observed. Compared to healthy older adults, those with T2DM demonstrated a similar capacity to adapt their postural control in response to a VST. However, this group exhibited different characteristic changes in sway which were linked to task performance.

